# The role of active music making in fostering resilience

**DOI:** 10.3389/fnins.2025.1629500

**Published:** 2025-08-26

**Authors:** Pauline Sophie Heck, Wiebke Reinhardt, Andrea Bischoff, Stefan Koelsch

**Affiliations:** ^1^Department of Biological and Medical Psychology, University of Bergen, Bergen, Norway; ^2^Independent Researcher, Essen, Germany

**Keywords:** musical sophistication, resilience, active music engagement, depression, emotion regulation, mental health

## Abstract

Previous studies suggest a positive association between engagement with music and psychological resilience, yet most existing research has focused on specific populations or isolated aspects of musical activity or resilience. The present study addresses this gap by examining the relationship between musical sophistication and resilience in a diverse adult sample recruited across several countries. An online survey was conducted with 392 healthy participants and 84 individuals with depression. Musical sophistication was assessed with the Goldsmiths Musical Sophistication Index (Gold-MSI), and resilience was measured using two validated scales (CD-RISC and BRS). Partial correlations in the healthy sample showed that musical sophistication was positively associated with resilience as measured by the CD-RISC, but not by the BRS. Contrary to the notion that individuals with depression may benefit less from music as a resilience resource due to maladaptive engagement patterns, we observed a positive – and even stronger – correlation with both resilience measures in this group. Further analyses revealed that among healthy individuals, active musical behaviours (such as making music or dancing) were more strongly associated with resilience than passive engagement (listening only). Both healthy and depressive participants reported feeling calmer and more peaceful after using music to cope with stress; this effect was significantly more frequent in the depressive group, particularly in the context of coping with depressive symptoms. Although the correlational design precludes causal conclusions, the findings suggest that musical sophistication is positively related to resilience, in both healthy and depressive individuals. Active music engagement appears particularly relevant for resilience among healthy individuals, while music-based coping may provide a stronger resource for those with depression. These findings offer promising directions for future research and may inform clinical applications involving music as a resilience-supportive resource.

## Introduction

Music is known to exert a variety of health-promoting effects, including the reduction of stress and pain symptoms, facilitation of emotional expression and emotion regulation, and the strengthening of social bonds (MacDonald et al., 2013). These functions are particularly relevant during times of crisis or adversity, which most individuals face at some point in their lives. While some experience long-lasting psychological impairments after such events—potentially culminating in clinical disorders—others recover relatively quickly and return to previous levels of mental health and functioning. A psychological construct central to this variability is resilience (Bonanno, 2021). Key elements of resilience, such as emotion regulation and social support (Southwick et al., 2011; Netuveli et al., 2008), overlap substantially with the psychological domains influenced by music, making the relationship between music and resilience an important topic for empirical investigation.

### Resilience

As with many psychological constructs, no universally accepted definition of resilience exists (Masten and Reed, 2002). One widely used conceptualization by Johnston et al. (2015) and Wadi et al. (2020) describes resilience as a dynamic capacity to adapt effectively to stress and adversity while maintaining mental health and well-being. Resilience is typically positively associated with sociodemographic variables such as education, occupation, and income, and it tends to increase with age (Bonanno et al., 2007; Bonanno and Mancini, 2008; MacLeod et al., 2016). Given its association with a wide range of positive outcomes—higher quality of life, improved mental health, reduced depressive symptoms, increased longevity, and lower mortality risk—resilience is increasingly seen as a target for both prevention and intervention efforts (MacLeod et al., 2016).

### Musical sophistication

Müllensiefen et al. (2014) introduced the concept of musical sophistication, which encompasses a broad spectrum of musical behaviors—both active (e.g., singing, playing an instrument) and passive (e.g., listening). Crucially, their operationalization does not limit the construct to professional musicians but extends to non-musicians and lay individuals, thereby allowing the measurement of musical engagement across the general population. Prior research has shown that musical sophistication is associated with younger age and higher socioeconomic status, possibly reflecting greater access to music education and more flexible use of leisure time.

### Previous research

A growing body of literature suggests that music engagement can evoke positive emotional states (Croom, 2014; Murcia et al., 2009; Grape et al., 2002), reduce physiological arousal during stress (Folkman and Moskowitz, 2000), lower cortisol levels (Le Roux et al., 2007), and enhance the release of endogenous opioids and endorphins (Van Ree et al., 2000), all of which are relevant to stress and pain regulation systems (Fields, 2007). Meta-analytic evidence confirms that music interventions can influence cardiovascular and hormonal markers of stress such as heart rate, blood pressure, and cortisol, adrenaline, and noradrenaline levels (de Witte et al., 2020). Additionally, anthropological and ethnomusicological studies have long emphasized the social bonding effects of music, highlighting its role in reinforcing social cohesion across cultures and historical periods (Tarr et al., 2014).

Given that emotion regulation, stress modulation, and social support are core components of resilience, it stands to reason that musical engagement might be associated with resilience-related outcomes. Accordingly, the present study investigates whether musical sophistication—as a multidimensional indicator of music engagement—is associated with resilience. Based on prior research, we predicted a positive association between musical sophistication and resilience, particularly in healthy individuals.

While earlier studies have hinted at such a relationship (Felsenstein, 2013; Gerber et al., 2014; Ajorpaz et al., 2014; Robarts, 2006; Rosenberg et al., 2021; Parker et al., 2021; Gupta and Singh, 2020), many have relied on narrow samples (e.g., clinical populations, trauma survivors), or used proxies of either musical engagement (e.g., listening frequency) or resilience (e.g., general well-being). By contrast, the present study used well-validated psychometric instruments to assess musical sophistication and resilience in a large, diverse, and international sample comprising both healthy individuals and those with mental or physical health conditions.

A recent study by our group (Koehler et al., 2023) further suggested that the mode of engagement—active versus passive—may moderate the relationship between music and resilience. Specifically, time spent playing music was positively associated with resilience outcomes, whereas time spent listening showed inconsistent or even negative associations (e.g., with optimism). We therefore hypothesized that the association between musical sophistication and resilience would be stronger among individuals who actively engage with music (e.g., making music, dancing) compared to those who engage passively (e.g., listening only).

### Resilience and music in depression

Individuals with depression or dysthymia may engage with music differently than healthy individuals, both in terms of listening preferences and emotional outcomes (Millgram et al., 2015; Yoon et al., 2020; Yoon and Rottenberg, 2020). For example, depressive individuals are more likely to select music that mirrors their affective state or enables emotional expression, whereas non-depressed individuals often choose music to enhance energy and inspiration (Wilhelm et al., 2013). Moreover, individuals experimentally induced into a sad mood tend to judge happy music as inappropriate or ineffective for mood improvement (Friedman et al., 2012). While some individuals with depression report seeking relaxation through sad music, they may also have reduced capacity to shift from negative to positive emotional states through music (Millgram et al., 2015; Yoon et al., 2020).

Given this potentially maladaptive engagement pattern, we hypothesized that depressive individuals would not benefit from musical sophistication to the same extent as healthy individuals in terms of its association with resilience. However, this question remains open and was empirically addressed in the present study.

## Materials and methods

### Participants

A total of 620 individuals (aged 18–90 years; M = 37.00, SD = 15.46; 419 males, 198 females, 3 identifying as other gender) completed the online survey in full. All participants confirmed that they understood the questions and responded honestly. The majority either held a university degree (53.1%) or were currently pursuing university studies (26.0%). Approximately one-third of participants resided in Norway, another third in Germany, and the remaining third lived in various countries across Europe, North and South America, Asia, and Australia.

No participants were excluded based on response speed, as none fell more than two standard deviations below the mean response time—a threshold used to screen for potentially insincere responding. However, 17 individuals were excluded due to indications of musical anhedonia, a condition characterized by a lack of pleasure and emotional response to music (Mas-Herrero et al., 2014; Holm et al., 2020).

To ensure a homogenous healthy sample for correlational analyses, we further excluded individuals with chronic physical illness. Although research on the effect of chronic pain on music engagement is scarce, preliminary findings suggest that chronic pain can reduce musical involvement and enjoyment (Gold and Clare, 2013), potentially influencing the association between musical sophistication and resilience. We also excluded individuals reporting mental illness other than depression, as existing literature does not yet provide a clear rationale for including these groups in analyses focused on music and resilience. Individuals reporting both physical and mental illness were likewise excluded. After these exclusions, the healthy sample consisted of 392 participants.

The depressive sample comprised 84 individuals, including participants who either self-reported a formal diagnosis of depressive disorder or met the diagnostic threshold on the Patient Health Questionnaire for Depression (PHQ-2) (Kroenke and Spitzer, 2002). This sample also included individuals with comorbid physical or mental illnesses.

### Ethics statement

The study was conducted in accordance with the principles of the Declaration of Helsinki. All participants provided informed consent prior to participation. Students from Heinrich Heine University received course credit in exchange for participation; no other compensation was offered.

### Questionnaire measures

The full survey, including item wordings, translation details, exclusion criteria, and descriptive statistics of questionnaire measurements, is provided in the Supplementary material.

#### Musical sophistication

Musical engagement was assessed using the Goldsmiths Musical Sophistication Index (Gold-MSI; 11), which measures both active and passive forms of musical behavior. To reduce survey length and participant fatigue—particularly critical in an online format—we administered a shortened version of the Gold-MSI, comprising 23 items from four subscales: “Active Engagement,” “Emotions,” “Musical Training,” and “Singing Abilities.” The “Perceptual Abilities” subscale was excluded to streamline the survey, a decision that is acknowledged as a potential source of measurement bias (see Discussion).

#### Musical anhedonia

Musical anhedonia was assessed using seven items from the Gold-MSI that closely resemble items from the Barcelona Music Reward Questionnaire (BMRQ) (Mas-Herrero et al., 2012), a validated instrument for identifying musical anhedonia. Participants scoring more than two standard deviations below the mean on these items were classified as musically anhedonic.

#### Resilience

Resilience was measured using two established instruments: the Connor-Davidson Resilience Scale (CD-RISC) (Connor and Davidson, 2003) and the Brief Resilience Scale (BRS) (Smith et al., 2008). The CD-RISC provides a multidimensional assessment of protective resilience factors such as emotion regulation, optimism, and competence, whereas the BRS focuses specifically on the individual’s perceived capacity to recover from stress (Smith et al., 2008). Together, these measures offer a more comprehensive picture of resilience.

#### Socio-economic status

Subjective socio-economic status (SES) was assessed using a questionnaire consisting of five items targeting education, training, occupation, income, and general social status, all rated relative to others in the participant’s country. In cases of cohabitation (e.g., with parents or partners), participants rated the highest income or occupational status in the household, following established recommendations (Hoebel et al., 2017). To allow comparison with more objective SES indicators, three additional items were included to assess school leaving certificate, highest professional qualification, and monthly net income. As with subjective SES, cohabitating individuals reported household income based on the highest-earning member.

#### Health status

Participants were asked whether they had been diagnosed with a chronic physical illness, with binary response options (“yes” or “no”). If “yes,” they were prompted to select one or more conditions from a predefined list. An analogous procedure was used to assess diagnosed mental illnesses.

#### Depression

In addition to asking directly about diagnoses of mental illness, the PHQ-2 (Kroenke and Spitzer, 2002) was administered to assess depressive symptoms. The PHQ-2 sum score ranges from 0 to 6, with scores ≥3 indicating probable major depression. To screen for a tendency toward recurrent depression, the timeframe of the questions was extended from 2 weeks to 5 years. A cutoff score of ≥5 on this adjusted scale was used to indicate a high likelihood of recurrent depressive episodes.

#### Use of music

Participants answered questions regarding their use of music in various contexts and for specific health conditions. The analysis focused on responses to a question about coping with stress, offering three options: listening to music, making music, or dancing. Additionally, participants who had reported experiencing a highly stressful event were asked how they typically felt after using music in such situations. Multiple response options were available, including “more encouraged/activated,” “more happy/cheerful,” “more calm/peaceful,” “rather demotivated,” and “sad.” Participants with diagnosed mental illness were also asked how they felt after using music specifically to cope with their illness.

The psychometric properties of all measures—Gold-MSI, musical anhedonia screening, CD-RISC, BRS, SES, and PHQ-2—ranged from acceptable (*α* = 0.70–0.80) to good (α = 0.80–0.90) to excellent (α > 0.90).

### Survey and procedure

The survey was implemented using the SoSci-Survey platform and was available in Norwegian, German, and English. Data collection ran from 19 February to 5 May 2021.

After selecting a preferred language, participants received information about eligibility (minimum age), data protection, voluntary participation, the right to withdraw at any time, and the estimated survey duration (10–15 min). The study was advertised as a survey on music and mood. After providing informed consent, participants completed sociodemographic questions and the questionnaires described above. They were then asked to confirm whether they had understood all questions and, if not, to indicate which items had been unclear. To assess data validity, participants were also asked whether they had answered honestly, as recommended by Meade and Craig (2012). Students from Heinrich Heine University could optionally provide their email address to receive course credit.

To mitigate potential order effects, the sequence of core questionnaires (including SES, CD-RISC, BRS, and Gold-MSI) was rotated after every 70 responses.

### Data analysis

This was an observational study analysed using IBM SPSS Statistics (Version 29.0.2.0). In the healthy sample (*n* = 392), one-tailed partial correlations were calculated to assess associations between musical sophistication and resilience. For the depressive sample (*n* = 84), two-tailed partial correlations were conducted. *Z*-standardized correlation coefficients from the two samples were then statistically compared.

To examine the role of active versus passive music use, participants were asked how they typically use music to cope with stress. Individuals who indicated making music and/or dancing were classified as active users, whereas those who indicated only listening to music were classified as passive users. Partial correlations between musical sophistication and resilience were calculated separately for each group, followed by statistical comparison of *Z*-standardized coefficients.

Given prior evidence linking SES and age to musical sophistication—and age and gender to resilience—partial correlations controlled for SES, age, and gender. This was supported by preliminary analyses showing significant associations: CD-RISC with age (*r* = 0.23, *p* ≤ 0.01), BRS with age (*r* = 0.23, *p* ≤ 0.01), BRS with gender (*r* = 0.14, *p* ≤ 0.01), and SES with CD-RISC, BRS, and Gold-MSI (*r* = 0.15, *p* < 0.001 for all; one-tailed). CD-RISC and BRS were also significantly correlated (*r* = 0.70, *p* < 0.001).

To compare resilience levels between healthy and depressive participants, independent-samples t-tests were conducted for both CD-RISC and BRS.

As the CD-RISC and BRS measure different facets of resilience, they were treated as independent variables in all analyses. The assumptions of interval scaling and linearity were met for all correlation analyses.

For an in-depth analysis, chi-square tests (*α* = 0.01) were used to compare frequencies of reported emotional outcomes following music use during highly stressful experiences. Because this question was only asked of participants who had reported such experiences, the number of respondents for this analysis differed from the total sample sizes.

## Results

The following results are described in detail and summarized in Table 1.

**Table 1:** Results of partial correlations in the healthy and depressive sample as well as for active and passive music use in the healthy sample.

Sample/Subsample		CD-RISC		BRS	
	*n*	*r*	*p*	*r*	*p*
Healthy participants	392	0.13^**^	0.005	0.03	0.278
Active music use	196	0.24^***^	<0.001	0.06	0.402
Passive music use	192	0.03	0.688	0.06	0.389
Depressive participants	84	0.23^*^	0.04	0.26^*^	0.018

^*^p ≤ 0.05, ^**^p ≤ 0.01, and ^***^p ≤ 0.001.

Among healthy participants, a small but statistically significant positive partial correlation was observed between musical sophistication and resilience, as measured by the CD-RISC [*r*(392) = 0.13, *p* = 0.005]. In contrast, no significant correlation emerged when resilience was measured with the BRS [*r*(392) = 0.03, *p* = 0.278].

When analyzing how participants used music to cope with stress, results indicated a significant association between musical sophistication and resilience (CD-RISC) for participants who engaged actively with music—i.e., through music-making or dancing [*r*(196) = 0.24, *p* < 0.001]. However, this association was not evident when resilience was assessed via BRS [*r*(196) = 0.06, *p* = 0.402]. In the group that used music passively—i.e., through listening only—no significant correlations were found for either CD-RISC [*r*(192) = 0.03, *p* = 0.688] or BRS [*r*(192) = 0.06, *p* = 0.389]. A comparison of the *Z*-standardized correlations for active versus passive music use (based on CD-RISC) yielded a significant difference (*Z* = 1.78, *p* = 0.037, one-tailed).

For participants with depressive disorder, significant positive correlations were found between musical sophistication and resilience as assessed by both instruments: CD-RISC [*r*(84) = 0.23, *p* = 0.040] and BRS [*r*(84) = 0.26, *p* = 0.018]. Comparing the *Z*-standardized coefficients between healthy and depressive participants showed no significant differences for either the CD-RISC (*Z* = −0.85, *p* = 0.397, two-tailed) or the BRS (*Z* = −1.96, *p* = 0.050, two-tailed). The sample of depressive participants was too small to permit reliable subgroup analyses based on active versus passive music use.

The independent samples *t*-tests revealed significant group differences in resilience. Healthy participants scored higher than depressive participants on both scales: CD-RISC (M = 1.83, SD = 0.52 vs. M = 1.41, SD = 0.56), *t*(474) = 6.680, *p* < 0.001, *d* = 0.53; and BRS (M = 3.52, SD = 0.66 vs. M = 2.73, SD = 0.77), *t*(111) = 8.788, *p* < 0.001, *d* = 0.68. Due to a violation of the assumption of homogeneity of variance for BRS, Welch’s correction was applied.

### In-depth analysis

In addition to the main analyses, participants were asked how they typically feel after using music to cope with a highly stressful experience. Response options were: “more encouraged/activated,” “more happy/cheerful,” “more calm/peaceful,” “rather demotivated,” and “sad.” Multiple selections were allowed. Both groups most frequently reported feeling more calm or peaceful, followed by feelings of activation, cheerfulness, sadness, and lastly, demotivation. A chi-square test showed that participants with depression were significantly more likely to report feeling calm or peaceful after using music to cope with stress compared to healthy participants [*χ*^2^(1) = 9.28, *p* = 0.002, *φ* = 0.17], indicating a small effect size. No other group differences reached statistical significance.

**Figure 1:**
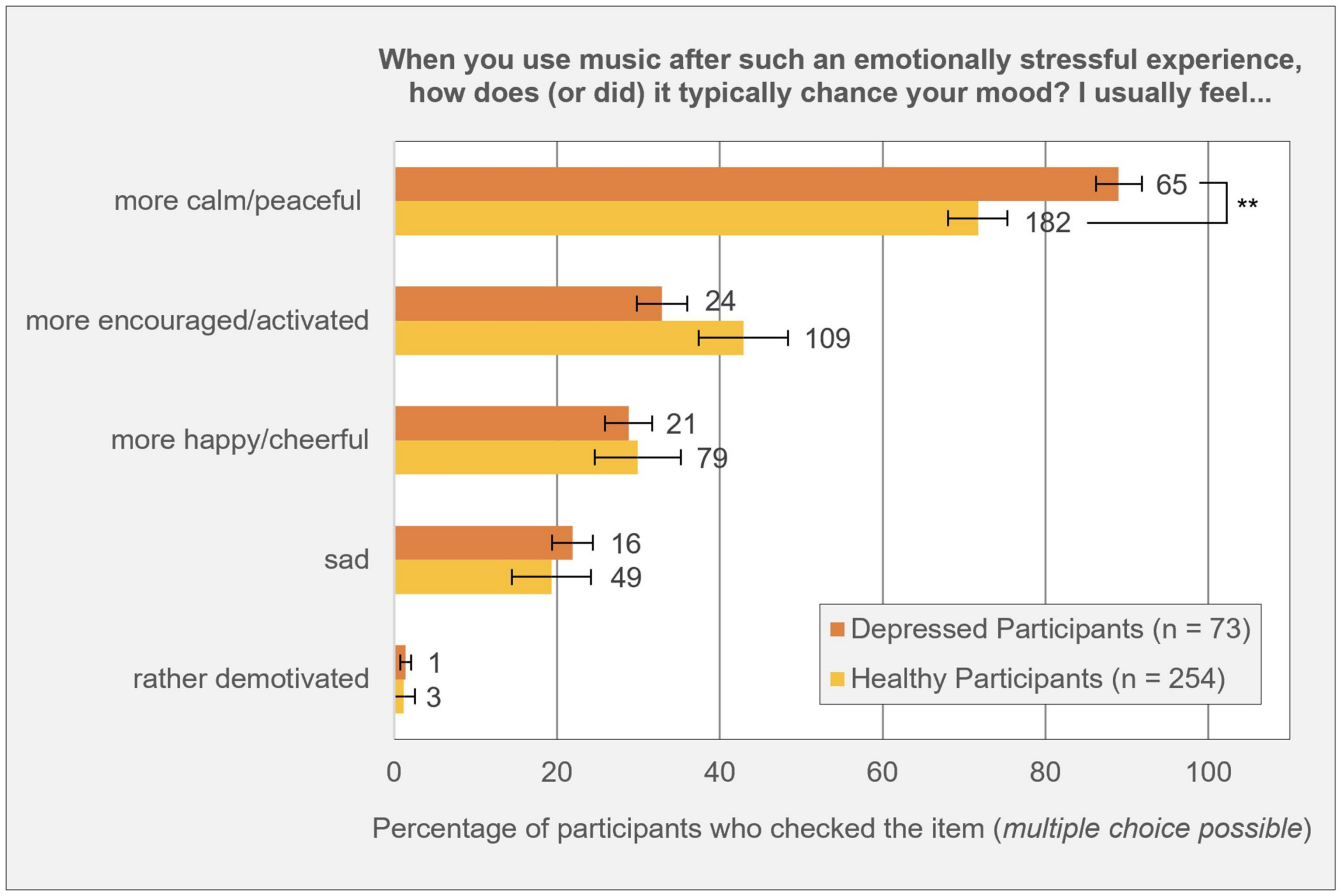
Change in mood in healthy and depressive participants after using music to cope with an emotionally stressful experience. ^**^p ≤ 0.01. Error bars indicate the standard error (SE) of the percentage. Number of absolute participants to the right of the bars.

Among those diagnosed with depression (*n* = 65) who used music to cope with their illness specifically, the same ranking of emotional effects was observed: 58 participants (89.2%) reported feeling more calm/peaceful, 28 (43.1%) reported feeling more encouraged/active, 24 (36.9%) felt more happy/cheerful, 14 (21.5%) felt sad, and only 3 (4.6%) reported feeling demotivated (see Figure 1).

## Discussion

Healthy participants showed a positive and significant correlation between musical sophistication and resilience as measured by the CD-RISC. Depressive participants exhibited positive and significant correlations between musical sophistication and resilience as measured by both the CD-RISC and the BRS. These findings indicate an association between musical sophistication and the protective factors for resilience (as measured by the CD-RISC) and perceived stress recovery ability (as measured by the BRS). Overall, these results support the notion of a relationship between musical sophistication and resilience. Considering previous research demonstrating associations between music engagement and improved emotion regulation, stress reduction, and social bonding—all factors linked to resilience—it is important to highlight that the present study’s correlational design precludes causal interpretations (MacDonald et al., 2013). Nevertheless, the correlation between CD-RISC scores and musical sophistication in both the healthy and depressive samples suggests that individuals who engage with music might have access to resources associated with coping more effectively with stress and possibly experiencing improved recovery outcomes. These correlational findings echo prior research indicating positive relationships between engagement with arts and enhanced health and wellbeing, including reduced depressive symptoms. Therefore, artistic engagement may be viewed as a potential protective factor associated with resilience (Fancourt et al., 2019; Fancourt and Finn, 2019).

The pattern of results observed for the two resilience scales warrants closer consideration. The CD-RISC and the BRS capture different facets of resilience: while the CD-RISC focuses on a multidimensional profile of protective factors (e.g., emotion regulation, optimism, personal competence), the BRS provides a more targeted assessment of perceived ability to recover from stress (“bouncing back”). In the present study, the BRS did not show significant correlations with musical sophistication in the healthy sample, whereas the CD-RISC did. This may indicate that in healthy individuals, musical engagement is more closely linked to a broad set of protective psychological traits than to the acute perception of stress recovery. In contrast, both scales showed significant correlations in the depressive sample, suggesting that for individuals with depression, musical sophistication may relate not only to protective factors but also to an enhanced perceived capacity for stress recovery. These differential findings illustrate the value of using multiple instruments to capture distinct but complementary components of resilience.

Within the healthy sample, analysing only participants who actively engaged with music (making music or dancing) for stress coping revealed an even stronger positive correlation between musical sophistication and resilience compared to the entire healthy sample. Conversely, examining only participants who passively engaged with music (listening only) after stressful experiences showed no significant correlation. The correlation coefficients of active versus passive groups differed significantly from each other. Although causality cannot be inferred, this finding indicates that active music engagement may be more strongly related to resilience compared to passive engagement in healthy individuals. This observation aligns with previous correlational findings by Koehler et al. (2023). However, this does not negate potential associations of passive music use with resilience. Given that the group of participants relying solely on music listening did not exhibit significant correlations, these individuals may preferentially use alternative coping resources such as physical activity, mindfulness techniques, relaxation strategies, spiritual or religious practices, or other hobbies. Future studies should explore in greater detail which coping strategies individuals predominantly use and how reliance on music specifically relates to resilience outcomes compared to other strategies.

It is also possible that healthy individuals, who typically have higher baseline resilience, possess more diverse coping strategies, potentially resulting in smaller correlations between musical sophistication and resilience. In contrast, individuals with depression, who generally show lower baseline resilience, might rely more heavily on music as a coping strategy. Given our correlational data and limited prior research, we emphasize that the reasons for these observed associations remain speculative. Nevertheless, these findings offer a promising foundation for future, more differentiated studies and have potential implications for clinical practice.

The in-depth analysis revealed that both depressive and healthy participants reported feeling calmer and more peaceful after using music for stress coping. Notably, depressive participants reported this significantly more frequently than healthy individuals. While this finding suggests music’s potential relevance as a resilience-related resource for individuals with depression, it is important to interpret it cautiously. The same musical piece might be perceived as “sad” by some listeners and “peaceful” by others (Taruffi and Koelsch, 2014), as music characterized as calm and peaceful can acoustically resemble music perceived as sad or melancholic. Therefore, depressive individuals and clinicians should carefully evaluate the emotional impact of music used therapeutically, as there remains a risk that certain music choices might inadvertently promote sadness and rumination (Garrido and Schubert, 2015; Koelsch et al., 2019; Koelsch et al., 2022).

### Limitations

The study sample exhibited a bias towards younger, male, and more educated participants. Additionally, participants were primarily residents of Norway and Germany, limiting the generalizability of findings to broader international populations. Moreover, the exclusion of the “Perceptual Abilities” subscale from the Gold-MSI might have biased our measure of musical sophistication toward active musical behaviours, potentially underrepresenting individuals who predominantly engage with music through listening. As a result, the observed relationships between musical sophistication and resilience could be less applicable to populations with primarily passive musical engagement.

Furthermore, the disparity in sample sizes between our healthy (*n* = 392) and depressive (*n* = 84) groups is an important characteristic of this study’s design. To clarify its impact, a *post hoc* power analysis was performed using G*Power 3.1.9.7. This confirmed that the study was excellently powered (>0.99) to detect the medium-to-large effect sizes reported here (CD-RISC: *d* = 0.53, power = 0.997; BRS: *d* = 0.68, power = 0.99997). However, it is important to note that the design was not sensitive enough to reliably detect smaller, more subtle between-group differences. Future research aiming to explore smaller effect sizes should therefore aim for more balanced group sizes to enhance sensitivity and interpretability.

Additionally, the online survey setting introduced uncontrolled variables, such as fluctuations in participants’ emotional states during survey completion, potentially influencing self-reported resilience. Finally, as this study relied exclusively on self-report questionnaires, responses could be influenced by social desirability and other biases inherent in self-assessment methodologies. Although honesty-check items and anonymity instructions were included, future studies should complement self-report data with objective or behavioural resilience measures. Alternative approaches, such as assessing individual flexibility in self-regulation, may also provide promising avenues for future research (Bonanno, 2021).

### Future directions

Our findings indicating associations between musical sophistication and resilience in both healthy and depressive individuals highlight the need for further research, particularly studies designed to establish causality. Future research should also explore populations with chronic physical illnesses, which were not adequately represented in our study. Given the small number of such participants, no conclusions could be drawn regarding musical sophistication’s potential association with resilience in these populations. Additionally, differentiating dancing and music-making separately, rather than collectively categorizing them as active music engagement, could provide more nuanced insights into how each activity individually relates to resilience.

## Conclusion

This study investigated associations between musical sophistication and resilience in healthy and depressive populations, finding positive correlations in both groups. Active music-making exhibited a particularly strong relationship with resilience among healthy individuals. While causal relationships cannot be established from correlational data, our findings highlight that engaging with music, particularly actively, might relate positively to resilience in both healthy and depressive individuals. Additionally, depressive individuals reported greater frequency of experiencing calmness and peacefulness after music-based stress coping than healthy individuals. Though seemingly beneficial, clinicians should carefully assess whether music perceived as calming also carries risks of inducing melancholic or sad feelings, potentially exacerbating depressive symptoms. As one of the first studies examining musical sophistication and resilience, these findings offer valuable insights for clinical applications and set the stage for future research.

## Data Availability

The original contributions presented in the study are included in the article/Supplementary material, further inquiries can be directed to the corresponding author.
