# Supplementary material for: The role of active music making in fostering resilience
Source: Front Neurosci. 2025 Aug 26;19:1629500. doi: 10.3389/fnins.2025.1629500 (PMC12418516; doi:10.3389/fnins.2025.1629500)
Supplement: Supplementary file 1 [file Data_Sheet_1.pdf]

S1 Appendix. Exclusion of items from the Gold-MSI.

To not exceed the intended survey length, the Gold-MSI questionnaire was reduced from 38 to 23 items across four subscales. The items were selected to match as closely as possible those of the Barcelona Music Reward Questionnaire (1) in order to be able to exclude participants with musical anhedonia. Another criterion for the reduction was to achieve at least an acceptable reliability for each subscale. Therefore, the reliabilities were calculated with a configuration tool on a website run by Müllensiefen and colleagues (2). Finally, items were chosen that were assumed to be as relevant as possible for all participants.

References

1. Mas-Herrero E, Marco-Pallares J, Lorenzo-Seva U, Zatorre RJ, Rodriguez-Fornells, A. Individual differences in music reward experiences. Music Percept. 2012;31(2):118-138.
2. GMSI Configurator [Internet]. London: Goldsmiths University of London [cited 2024 June 11]. Available from: https://shiny.gold-msi.org/gmsiconfigurator/
