# Supplementary material for: The role of active music making in fostering resilience
Source: Front Neurosci. 2025 Aug 26;19:1629500. doi: 10.3389/fnins.2025.1629500 (PMC12418516; doi:10.3389/fnins.2025.1629500)
Supplement: Supplementary file 2 [file Data_Sheet_2.pdf]

S2 Appendix. Complete Questionnaire in English.

Welcome and thank you for taking your time to participate in our study on music and moods under stress.
Participation is only allowed for individuals with a minimum age of 18 years.
The survey will take about 15 minutes. Please make sure that you are undisturbed during this time.
Your participation is voluntary, refusal to participate will not result in any negative consequences. You can terminate participation at any time (but we can evaluate your data only if you answer all questions). Your answers will be anonymized, so they cannot be assigned to you personally.
By continuing to the next page, you agree to these conditions.

Socio-demographic questions

First, we would like you to answer some demographic questions.

- SD01: How old are you?
    - I am ... years old.
- SD02: What is your gender?
    - Female
    - Male
    - Other
- SD03: In which country do you live?
    - I live in…

Subjective social status

In the following we would like you to answer some questions regarding your socio-economic situation.

- SE01: Compared with the citizens of your country: How do you rate your highest school leaving certificate (e. g. high school diploma, GCSE)?
    - Well above average
    - Slightly above average
    - Average
    - Slightly below average
    - Well below average
- SE02: Compared with the citizens of your country: How do you rate your highest professional training qualification or university degree? If you are still in school, training or studying, proceed from your expected training qualification.
    - Well above average
    - Slightly above average
    - Average
    - Slightly below average
    - Well below average
- SE03: Compared with the citizens of your country: How do you rate the net income of the household in which you live? If you are still in school, training or studying, rate the income of your realistically expected occupation.
    - Well above average
    - Slightly above average
    - Average
    - Slightly below average
    - Well below average
- SE04: Compared with the citizens of your country: How do you rate the social status of your job? If you live together with your partner, and the social status of his or her job is higher than yours, rate the status of your partner's job. If you are still in school, training or studying, assume the status of your realistically expected job. In the event of unemployment or time off work rate the social status of your last job.
    - Well above average

    - Slightly above average
    - Average
    - Slightly below average
    - Well below average
- SE05: Compared with the citizens of your country: How do you rate your overall social status? If you are still in school, training or studying, rate your realistically expected social status.
    - Well above average
    - Slightly above average
    - Average
    - Slightly below average
    - Well below average

Objective socio-economic status

- SE06: What is your highest school leaving certificate (e. g. high school diploma, GCSE, no school leaving certificate yet)?
- SE07: What is your highest professional qualification or university degree?
    - University degree
    - No university degree yet, but studying at university
    - Professional qualification with degree (not university)
    - No professional qualification yet, but currently in training for a professional qualification
    - Without any professional degree
    - Still in school
- SE08: How much is your monthly net income? Please also provide the currency. If you live together with a partner and your partner has a higher net income than you, state the net income of your partner. If you are still in education or studying, use the income of the highest-earning parent from the household in which you live or where you last lived.
    - My / my partner's / my parent's monthly net income (including currency):
    - I do not want to specify / I do not know

Musical sophistication

In the following, we will give you some statements and ask a few questions about your musical background, interests, and activities. Please tick or fill in the answer that applies to you the best.

- Scale: Completely disagree, strongly disagree, disagree, neither agree nor disagree, agree, strongly Agree, completely agree.
- MS02_01: I spend a lot of my free time doing music-related activities.
- MS02_02: I enjoy writing about music, for example on blogs and forums.
- MS02_03: I often read or search the internet for things related to music.
- MS02_04: I do not spend much of my disposable income on music.
- MS02_05: Music is kind of an addiction for me – I could not live without it.
- MS02_06: I keep track of new music that I come across (e.g. new artists or recordings).
- MS02_07: I sometimes choose music that can trigger shivers down my spine.
- MS02_08: Pieces of music rarely evoke emotions for me.
- MS02_09: I often pick certain music to motivate or excite me.
- MS02_10: I am able to identify what is special about a given musical piece.
- MS02_11: I am able to talk about the emotions that a piece of music evokes for me.
- MS02_12: Music can evoke my memories of past people and places.
- MS02_13: I have never been complimented for my talents as a musical performer.
- MS02_14: I would not consider myself a musician.
- MS02_15: I can sing or play music from memory.
- MS02_16: I am able to hit the right notes when I sing along with a recording.
- MS02_17: I am not able to sing in harmony when somebody is singing a familiar tune.

- MS03 (AE_08): I listen attentively to music for ... per day.
    - 0-15 min
    - 15-30 min
    - 30-60 min
    - 60-90 min
    - 2 hours
    - 2-3 hours
    - 4 hours or more
- MS04 (MT_01): I engaged in regular, daily practice of a musical instrument (including voice) for ... years.
    - 0 / 1 / 2 / 3 / 4-5 / 6-9 / 10 or more
- MS05 (MT_02): At the peak of my interest, I practised my primary instrument for ... hours per day.
    - 0 / 0,5 / 1 / 1,5 / 2 / 3-4 /5 or more
- MS06 (MT_04): I have had formal training in music theory for ... years.
    - 0 / 0,5 / 1 / 2 / 3 /4-6 / 7 or more
- MS07 (MT_05): I have had ... years of formal training on a musical instrument (including voice) during my lifetime.
    - 0 / 0,5 / 1 / 2 / 3-5 / 6-9 / 10 or more
- MS08 (MT_06): I can play ... musical instruments.
    - 0 / 1 / 2 / 3 / 4 / 5 / 6 or more

Resilience

Now we would like you to answer a few more questions about yourself. Please indicate how much you agree with each of the following statements.

- RS02: CD_RISC
  Scale: Not true at all, rarely true, sometimes true, often true, true nearly all the time
    - RS02_01: I am able to adapt when changes occur.
    - RS02_02: I can deal with whatever comes my way.
    - RS02_03: I try to see the humorous side of things when I am faced with problems.
    - RS02_04: Having to cope with stress can make me stronger.
    - RS02_05: I tend to bounce back after illness, injury, or other hardships.
    - RS02_06: I believe I can achieve my goals, even if there are obstacles.
    - RS02_07: Under pressure, I stay focused and think clearly.
    - RS02_08: I am not easily discouraged by failure.
    - RS02_09: I think of myself as a strong person when dealing with life's challenges and difficulties.
    - RS02_10: I am able to handle unpleasant or painful feelings like sadness, fear and anger.
- RS03: BRS
  Scale: Strongly disagree, disagree, neutral, agree, strongly agree
    - RS03_01: I tend to bounce back quickly after hard times.
    - RS03_02: I have a hard time making it through stressful events.
    - RS03_03: It does not take me long to recover from a stressful event.
    - RS03_04: It is hard for me to snap back when something wrong happens.
    - RS03_05: I usually come through difficult times with little trouble.
    - RS03_06: I tend to take a long time to get over setbacks in my life.

Use of music

Please answer a few questions about the use of music in your everyday life.

- MU02: When I am stressed, I listen to or play music to calm myself or to feel better.
    - Never
    - Rarely
    - Sometimes

    - Often
    - Very often
- MU03: How do you use music to cope with stress? I usually... (multiple answers possible)
    - MU03_ 01: Listen to music
    - MU03_ 02: Make music
    - MU03_ 03: Dance
- MU04: Have you ever had an emotionally highly stressful experience in your life, e. g. abuse / extreme violence / witness a disaster / divorce / mobbing / serious accident / serious illness / loss of a close relative?
    - Yes
    - No
- MU05: After such an emotionally stressful experience, have you used music to cope with it, e.g to comfort, distract or motivate yourself?
    - Not at all
    - A little bit
    - Partially
    - Mostly
    - Very much
- MU06: How did (or do) you use music to cope with such an emotionally stressful experience? I usually ... (multiple answers are possible)
    - MU06_01: Listen to music
    - MU06_02: Make music (e. g. instrument, vocals, composing)
    - MU06_03: Dance
- MU07: When you use music after such an emotionally stressful experience, how does (or did) it typically change your mood? I usually feel ... (multiple answers are possible)
    - MU07_01: More encouraged / activated
    - MU07_02: More happy / cheerful
    - MU07_03: More calm / peaceful
    - MU07_04: Rather demotivated
    - MU07_05: Sad
    - MU07_06: Other, please specify:...
- MU08: During the Covid-19 pandemic, some people use music to reduce their psychological stress. Does this also apply to you?
    - Not at all
    - A little bit
    - Partially
    - Mostly
    - Very much

Size and weight

- MU09: How tall are you?
    - I am … cm tall.
- MU10: How much do you weigh?
    - I weigh … kg.

Chronic physical illness and mental illness

- MV01: Do you have a diagnosed chronic physical illness e. g. chronic back pain, diabetes or other?
    - Yes
    - No
- MV02: Which one/s?
    - MV02_01: Cardiovascular / heart disease
    - MV02_02: Cancer
    - MV02_03: Chronic respiratory disease

- MV02_04: Diabetes
- MV02_05: Dementia
- MV02_06: Autoimmune disease
- MV02_07: Chronic back pain
- MV02_08: other, please specify: …

- MV03: Some people with a chronic illness use music to cope with it e.g., to comfort, distract or motivate themselves. Does this also apply to you?
    - Not at all
    - A little bit
    - Partially
    - Mostly
    - Very much
- MV04: How do you use music to cope with your chronic illness? I usually… (multiple answers are possible)?
    - MV04_01: Listen to music
    - MV04_02: Make music (e. g. instrument, vocals, composing)
    - MV04_03: Dance
- MV05: When you use music to cope with your chronic illness, how does (or did) it typically change your mood? I usually … (multiple answers are possible)?
    - MV05_01: More encouraged / activated
    - MV05_02: More happy / cheerful
    - MV05_03: More calm / peaceful
    - MV05_04: Rather demotivated
    - MV05_05: Sad
    - MV05_06: Other, please specify…
- MV06: Do you have a diagnosed mental disorder e.g., depression or anxiety disorder?
    - Yes
    - No
- MV07: Which ones?
    - MV07_1: Depression disorder
    - MV07_2: Bipolar disorder
    - MV07_3: Anxiety disorder
    - MV07_4: Obsessive-compulsive disorder
    - MV07_5: Post Traumatic Stress Disorder
    - MV07_6: Addiction or substance abuse disorder (e.g., addiction to alcohol, cannabis, hallucinogens, sedatives)
    - MV07_7: Schizophrenia
    - MV07_8: Eating disorder
    - MV07_9: Other, please specify
- MV08: Some people with a mental disorder use music to cope with it e.g., to comfort, distract or motivate themselves.
    - Not at all
    - A little bit
    - Partially
    - Mostly
    - Very much
- MV09: How do you use music to cope with your mental disorder? I usually…(multiple answers are possible)
    - MV09_01: Listen to music
    - MV09_02: Make music (e. g. instrument, vocals, composing)
    - MV09_03: Dance

- MV10: When you use music to cope with your mental disorder, how does (or did) it typically change your mood? I usually … (multiple answers are possible)?
    - MV10_01: More encouraged / activated

    - MV10_02: More happy / cheerful
    - MV10_03: More calm / peaceful
    - MV10_04: Rather demotivated
    - MV10_05: Sad
    - MV10_06: Other, please specify…

PHQ-2

We are nearly done! Now a few questions regarding your mood.
Please indicate how much you agree with each of the following statements!

- DE02: Major depression: Over the past 2 weeks, how often have you been bothered by any of the following problems?
- Scale: Not at all, several days, more than half of the days, almost every day
    - DE02_01: I had little interest or pleasure in doing things.
    - DE02_02: I felt down, depressed, or hopeless.
- DE03: Recurrent depression: Over the past 5 years, how often have you been bothered during a period of two weeks or more by any of the following problems?
- Scale: Never, during one year, during several years, during every year
    - DE03_01: I had little interest or pleasure in doing things (for a period of at least two weeks).
    - DE03_02: I felt down, depressed, or hopeless (for a period of at least two weeks).

Section EN

We are at the end now, well done!

- EN01: Did you understand the questions in this survey?
    - Yes, I understood all of them.
    - No, I have not understood one or two questions. Could you specify which one/s?
    - No, I have not understood several or many questions.
- EN02: And last: Did you answer all questions honestly and to the best of your knowledge? Please answer this question honestly, you will not suffer any disadvantages.
    - Yes, I answered all questions to the best of my knowledge.
    - No

Thank you very much for your participation and your help in our research.
If you are interested in the results monitor tweets from @StefanKoelsch on Twitter.
This survey contains questionnaires for which all rights are reserved. Therefore, no part of this survey may be reproduced or transmitted in any form, or by any means, electronic or mechanical, including photocopying, or by any information storage or retrieval system, without permission in writing from Prof. Dr. Stefan Koelsch (stefan.koelsch@uib.no) or one of his colleagues associated with this project.
This survey also contains questions from the Goldsmiths Musical Sophistication Index (Gold-MSI) which are freely available under https://www.gold.ac.uk/music-mind-brain/gold-msi/.
