# Supplementary material for: The role of active music making in fostering resilience
Source: Front Neurosci. 2025 Aug 26;19:1629500. doi: 10.3389/fnins.2025.1629500 (PMC12418516; doi:10.3389/fnins.2025.1629500)
Supplement: Supplementary file 3 [file Data_Sheet_3.pdf]

S3 Appendix. Complete Questionnaire in Norwegian.

Velkommen og takk for at du tar deg tid til å delta i vårt studie om musikk og humør under stress. Deltakelse er kun godkjent for individer som er 18 år eller eldre. Spørreundersøkelsen tar omtrent 15 minutter. Vennligst pass på at du er uforstyrret under besvarelsen. Din deltakelse er frivillig, og om du nekter å besvare vil det ikke medføre negative konsekvenser. Du kan avslutte deltakelsen når som helst (men vi kan kun evaluere dataen din hvis du besvarer alle spørsmålene). Svarene dine er anonyme, så de kan ikke knyttes til deg personlig. Ved å fortsette til neste side godtar du disse betingelsene.

Socio-demographic questions

Først vil vi at du skal svare på noen demografiske spørsmål.

- SD01: Hvor gammel er du?
    - Jeg er … år gammel.
- SD02: Hvilket kjønn er du?
    - Kvinne
    - Mann
    - Annet
- SD03: Hvilket land bor du i?
    - Jeg bor i…

Subjective social status

I følgende del ønsker vi at du besvarer noen spørsmål om din sosio-økonomiske situasjon.

- SE01: Sammenlignet med innbyggere i landet ditt: Hvordan vil du rangert ditt høyeste vitnemål (for eksempel vitnemål fra videregående/ungdomsskolen)?
    - Langt over gjennomsnittet
    - Litt over gjennomsnittet
    - Gjennomsnittlig
    - Litt under gjennomsnittet
    - Langt under gjennomsnittet
- SE02: Sammenlignet med innbyggere i landet ditt: Hvordan vil du rangere din høyeste universitetsgrad eller profesjonelle opplæringskvalifikasjon? Dersom du fortsatt går på skole, er under opplæring eller studerer, ranger ut fra din forventede kvalifikasjon.
    - Langt over gjennomsnittet
    - Litt over gjennomsnittet
    - Gjennomsnittlig
    - Litt under gjennomsnittet
    - Langt under gjennomsnittet
- SE03: Sammenlignet med innbyggere i landet ditt: Hvordan vil du rangere din årlige nettolønn til husstanden du bor i? Dersom du fortsatt går på skole, er under opplæring eller studerer, ranger inntekten av din realistisk forventede ansettelse.
    - Langt over gjennomsnittet
    - Litt over gjennomsnittet
    - Gjennomsnittlig
    - Litt under gjennomsnittet
    - Langt under gjennomsnittet
- SE04: Sammenlignet med innbyggere i landet ditt: Hvordan rangerer du den sosiale statusen av jobben din? Dersom du bor sammen med en partner, og den sosiale statusen av hennes eller hans jobb er høyere enn din egen, ranger statusen av din partner sin jobb. Dersom du enda går på skole, er under opplæring eller studerer, anta statusen av din realistisk forventede jobb. Dersom du er arbeidsledig eller du har en pause fra jobb, ranger den sosiale statusen av din forrige jobb.
    - Langt over gjennomsnittet
    - Litt over gjennomsnittet

    - Gjennomsnittlig
    - Litt under gjennomsnittet
    - Langt under gjennomsnittet
- SE05: Sammenlignet med innbyggere av landet ditt: Hvordan vil du rangere din sosiale status? Dersom du fortsatt går på skole, er under opplæring eller studerer, ranger din realistisk forventede sosiale status.
    - Langt over gjennomsnittet
    - Litt over gjennomsnittet
    - Gjennomsnittlig
    - Litt under gjennomsnittet
    - Langt under gjennomsnittet

Objective socio-economic status

- SE06: I hvilke høyeste grad har du mottatt vitnemål (for eksempel videregående eller ungdomsskole)?
- SE07: Hva er din høyeste profesjonelle kvalifikasjon eller universitetsgrad?
    - Grad fra universitet eller høyskole
    - Holder på med å ta en grad ved universitet eller høyskole
    - Profesjonell kvalifikasjon med grad (ikke universitet)
    - Ingen profesjonell kvalifikasjon ennå, men for tiden i opplæring for en profesjonell kvalifikasjon.
    - Uten en grad
    - Går fortsatt på videregående eller ungdomsskole
- SE08: Hvor mye er din månedlige nettoinntekt? Vennligst oppgi valuta. Dersom du bor sammen med en partner og partneren din har høyere nettoinntekt enn deg, oppgi partneren sin nettoinntekt. Dersom du er under utdanning, oppgi inntekten til en forelder med høyest mulig inntekt i din husstand eller i din forrige husstand.
    - min / min partners / foreldres nettoinntekt (inkludert valuta):
    - Jeg vil ikke spesifisere / jeg vet ikke

Musical sophistication

I den følgende delen vil vi legge frem noen påstander og spørre noen få spørsmål om din musikalske bakgrunn, interesser og aktiviteter. Vennligst kryss av eller fyll inn svarene som passer deg best.

- Skala: Fullstendig uenig, meget uenig, uenig, verken enig eller uenig, enig, meget enig, fullstendig enig.
- MS02_01: Jeg bruker mye av min tid på musikkrelaterte aktiviteter.
- MS02_02: Jeg liker godt å skrive om musikk, for eksempel på blogger og internettforum.
- MS02_03: Jeg leser ofte om eller søker på internett etter ting som er relatert til musikk.
- MS02_04: Jeg bruker ikke mye penger på musikk.
- MS02_05: Det er som om jeg er avhengig av musikk – jeg kunne ikke levd uten.
- MS02_06: Jeg følger med på ny musikk som jeg støter på (f.eks nye artister eller innspillinger).
- MS02_07: Jeg velger ofte å lytte til musikk som kan gi meg gåsehud.
- MS02_08: Musikkstykker fremkaller sjeldent følelser i meg.
- MS02_09: Jeg velger ofte ut bestemt musikk for å motivere eller stimulere meg.
- MS02_10: Jeg er I stand til å identifisere hva som er spesielt ved et bestemt musikkstykke.
- MS02_11: Jeg er i stand til å snakke om følelsene et musikkstykke fremkaller i meg.
- MS02_12: Som regel vekker musikk minner om mennesker jeg har kjent eller steder jeg har vært.
- MS02_13: Jeg har aldri fått ros for mine talenter som musiker.
- MS02_14: Jeg betrakter ikke meg selv som en musiker.
- MS02_15: Jeg kan synge eller spille musikk utenat.
- MS02_16: Jeg er i stand til å treffe riktige toner når jeg synger med på et musikkopptak.
- MS02_17: Jeg er ikke i stand til å synge i harmoni når noen synger en velkjent melodi.

- MS03 (AE_08): Jeg lytter oppmerksomt til musikk … om dagen.
    - 0-15 min
    - 15-30 min
    - 30-60 min
    - 60-90 min
    - 2 timer
    - 2-3 timer
    - 4 eller flere timer
- MS04 (MT_01):. Jeg har regelmessig og daglig øvet på et musikkinstrument (inkludert sangstemme) i … år
    - 0 / 1 / 2 / 3 / 4-5 / 6-9 / 10 eller flere
- MS05 (MT_02): Da min interesse var på sitt høyeste, øvet jeg på mitt primære instrument … timer om dagen.
    - 0 / 0,5 / 1 / 1,5 / 2 / 3-4 /5 eller fler
- MS06 (MT_04): Jeg har fått undervisning i musikkteori i … år (utenom obligatorisk skole undervisning)
    - 0 / 0,5 / 1 / 2 / 3 /4-6 / 7 eller fler
- MS07 (MT_05): I løpet av livet har jeg fått undervisning i å spille et musikkinstrument (inkludert sangstemmen) i … år.
    - 0 / 0,5 / 1 / 2 / 3-5 / 6-9 / 10 eller fler
- MS08 (MT_06): Jeg kan spille … musikkinstrumenter.
    - 0 / 1 / 2 / 3 / 4 / 5 / 6 eller fler

Resilience

Nå ønsker vi at du svarer på noen få spørsmål om deg selv. Vennligst indiker hvor mye du er enig med hver av de følgende påstandene.

- RS02: CD_RISC
  Skala: Ikke sant i det hele tatt, sjeldent sant, av og til sant, ofte sant, nesten alltid sant.
    - RS02_01: Jeg klarer å tilpasse meg når det skjer forandringer.
    - RS02_02: Jeg kan håndtere de fleste ting som kommer min vei.
    - RS02_03: Når jeg står overfor problemer prøver jeg å se de humoristiske sidene ved ting.
    - RS02_04: Det å måtte takle stress kan gjøre meg sterkere.
    - RS02_05: Jeg pleier å komme meg etter sykdom, skader eller andre påkjenninger.
    - RS02_06: Jeg tror jeg kan oppnå mine mål, selv om det er hindringer i veien.
    - RS02_07: Under press er jeg fokusert og tenker klart.
    - RS02_08: Jeg blir ikke lett motløs av nederlag.
    - RS02_09: Jeg ser meg selv som en sterk person når det gjelder å takle livets utfordringer og vanskeligheter.
    - RS02_10: Jeg kan håndtere ubehagelige eller vonde følelser som tristhet, frykt eller sinne.
- RS03: BRS
  Skala: Veldig uenig, uenig, nøytral, enig, veldig enig
    - RS03_01: Jeg har en tendens til å komme meg fort etter vanskelige tider
    - RS03_02: Jeg sliter med å komme meg gjennom stressende hendelser.
    - RS03_03: Det tar meg ikke lang tid å komme meg etter en stressende hendelse.
    - RS03_04: Det er vanskelig for meg å komme meg når noe går galt.
    - RS03_05: Jeg kommer meg vanligvis gjennom vanskelige tider med lite problem.
    - RS03_06: Jeg har en tendens til å bruke lang til på å overkomme motgang i livet.

Use of music

Svar på noen spørsmål om bruk av musikk i hverdagen din.

- MU02: Når jeg er stresset lytter jeg til eller spiller musikk for å roe meg selv ned eller for å føle meg bedre.

- Aldri
- Sjeldent
- Av og til
- Ofte
- Veldig ofte

- MU03: Hvordan bruker du musikk for å takle stress? Jeg pleier å ... (flere mulige svar)
  - MU03_ 01: Høre på musikk
  - MU03_ 02: Lage musikk (synge, spille instrument, komponere)
  - MU03_ 03: Danse
- MU04: Har du noen gang hatt en følelsesmessig veldig stressende opplevelse i livet ditt, f.eks. overgrep / ekstrem vold / vitne til en katastrofe / skilsmisse / mobbing / alvorlig ulykke / alvorlig sykdom / tap av en nær slektning?
  - Ja
  - Nei
- MU05: Etter en så følelsesmessig stressende opplevelse, har du brukt musikk til å takle den, for eksempel for å trøste eller motivere deg selv?
  - Ikke i det hele tatt
  - Litt
  - Delvi
  - For det meste
  - Mye
- MU06: Hvordan brukte (eller bruker) du musikk for å takle en slik følelsesmessig stressende opplevelse? Jeg pleier ... (flere svar er mulig)
  - MU06_01: Hører jeg på musikk
  - MU06_02: Lager jeg musikk (for eksempel, instrument, vokal, komponere)
  - MU06_03: Danser jeg
- MU07: Når du bruker musikk etter en slik følelsesmessig stressende opplevelse, hvordan endrer (eller endret) det vanligvis humøret ditt? Jeg føler meg vanligvis ... (flere svar er mulige)
  - MU07_01: Mer oppmuntret / energis
  - MU07_02: Mer glad / munter
  - MU07_03: Mer rolig / fredfull
  - MU07_04: Demotivert
  - MU07_05: Trist
  - MU07_06: Andre, vennligst spesifiser…
- MU08: Under Covid-19 pandemien benytter noen musikk for å redusere sitt psykiske stress. Gjelder dette deg?
  - Ikke i det hele tatt
  - Litt
  - Delvis
  - For det meste
  - Mye

Size and weight

- MU09: Hvor høy er du?
  - Jeg er … cm høy.
- MU10: Hvor mye veier du?
  - Jeg veier … kg.

Chronic physical illness and mental illness

- MV01: Har du en diagnostisert kronisk fysisk sykdom (f. eks. kroniske ryggplager, diabetes eller annet)?
  - Ja
  - Nei

- MV02: Isåfall, hvilke?
    - MV02_01: Kardiovaskulær / hjertesykdom
    - MV02_02: Kreft
    - MV02_03: Kronisk luftveissykdom
    - MV02_04: Diabetes
    - MV02_05: Demens
    - MV02_06: Autoimmun sykdom
    - MV02_07: Kronisk ryggsmerter
    - MV02_08: Andre, vennligst spesifiser...
- MV03: Noen mennesker med kronisk sykdom bruker musikk for å takle situasjonen, for eksempel ved å berolige, distrahere eller motivere seg selv. Gjelder dette deg også?
    - Ikke i det hele tatt
    - Litt
    - Delvis
    - For det meste
    - Mye
- MV04: Hvordan benytter du musikk for å takle din kroniske sykdom? Vanligvis …(det er mulig å svare på flere).
    - MV04_01: Hører jeg på musikk
    - MV04_02: Lager jeg musikk (for eksempel, instrument, vokal, komponere)
    - MV04_03: Danser jeg
- MV05: Når du bruker musikk for å takle din kroniske sykdom, hvordan endrer (eller endret) den humøret ditt? Jeg føler meg vanligvis … (mulig med flere svar)
    - MV05_01: Mer oppmuntret / energisk
    - MV05_02: Mer glad / munter
    - MV05_03: Mer rolig / fredfull
    - MV05_04: Demotivert
    - MV05_05: Trist
    - MV05_06: Andre, vennligst spesifiser…
- MV06: Har du en diagnostisert mental forstyrrelse, for eksempel depresjon eller angstlidelse?
    - Ja
    - Nei
- MV07: Isåfall, hvilke/n?
    - MV07_1: Depresjon
    - MV07_2: Bipolar lidelse
    - MV07_3: Angst lidelse
    - MV07_4: Tvangstanker
    - MV07_5: Posttraumatisk stresslidelse
    - MV07_6: Avhengighet eller rusmisbruk (eks. Avhengig av alkohol, cannabis, hallusinogener, beroligende midler)
    - MV07_7: Schizofreni
    - MV07_8: Spiseforstyrrelser
    - MV07_9: Andre, vennligst spesifiser...
- MV08: Noen mennesker med en mental forstyrrelse bruker musikk for å takle den, for eksempel for trøst, distraksjon eller for å motivere seg selv.
    - Ikke i det hele tat
    - Litt
    - Delvis
    - For det meste
    - Mye

- MV09: Hvordan benytter du musikk for å takle din mentale forstyrrelse? Vanligvis…(det er mulig å svare på flere).
    - MV09_01: Hører jeg på musikk
    - MV09_02: Lager jeg musikk (for eksempel, instrument, vokal, komponere)

- MV09_03: Danser jeg
- MV10: Når du bruker musikk for å takle din mentale forstyrrelse, hvordan endrer (eller endret) den humøret ditt? Jeg føler meg vanligvis … (mulig med flere svar)
    - MV10_01: Mer oppmuntret/energisk
    - MV10_02: Mer glad/munter
    - MV10_03: Mer rolig/fredfull
    - MV10_04: Demotivert
    - MV10_05: Trist
    - MV10_06: Andre, vennligst spesifiser…

PHQ-2

Vi er nesten ferdig! Nå kommer noen få spørsmål angående humør. Vennligst indiker hvor mye du er enig med hver av de følgende påstandene!

- DE02: Over de siste 2 ukene, hvor ofte har du vært plaget av noen av de følgende problemene?
- Skala: Ikke i det hele tatt, flere dager, mer enn halvparten av dagene, nesten hver dag
    - DE02_01: Jeg hadde lite interesse eller glede av å gjøre ting.
    - DE02_02: Jeg følte meg nede, deprimert eller håpløs.
- DE03: I løpet av de siste 5 årene, hvor ofte har du vært plaget over en periode på to uker eller mer av noen av de følgende problemene?
- Skala: Aldri, i løpet av ett år, i flere år, i løpet av hvert år
    - DE03_01: Jeg hadde liten interesse eller glede av å gjøre ting (i en periode på minst to uker).
    - DE03_02: Jeg følte meg nedstemt, deprimert eller håpløs (i en periode på minst to uker).

Section EN

Vi er kommet til slutten nå, bra jobbet!

- EN01: Forsto du spørsmålene i denne undersøkelsen?
    - Ja, jeg forsto alle.
    - Nei, det er ett eller to spørsmål jeg ikke har forstått. Kan du spesifisere hvilket eller hvilke?
    - Nei, det er flere spørsmål jeg ikke har forstått.
- EN02: Og til slutt: Svarte du på alle spørsmålene ærlig og etter beste kunnskap? Vennligst svar på dette spørsmålet ærlig, det vil ikke medføre noen ulemper for deg.
    - Ja, jeg svarte på alle spørsmål så godt jeg kunne.
    - Nei

Tusen takk for din deltakelse og din hjelp i forskningen vår.
Hvis du er interessert i resultatene, kan du følge med på tweets fra @StefanKoelsch på Twitter.
Denne undersøkelsen inneholder spørreskjemaer hvor alle rettigheter er forbeholdt. Derfor kan ingen deler av denne undersøkelsen reproduseres eller overføres i noen form, eller på noen måte, elektronisk eller mekanisk, inkludert fotokopiering, eller med noe informasjonslagrings- eller gjenopprettingssystem, uten skriftlig tillatelse fra professor Dr. Stefan Koelsch (stefan.koelsch @ uib.no) eller en av hans kolleger tilknyttet dette prosjektet. Denne undersøkelsen inneholder også spørsmål fra Goldsmiths Musical Sophistication Index (Gold-MSI) som er fritt tilgjengelig under https://www.gold.ac.uk/music-mind-brain/gold-msi/.
