# Supplementary material for: The role of active music making in fostering resilience
Source: Front Neurosci. 2025 Aug 26;19:1629500. doi: 10.3389/fnins.2025.1629500 (PMC12418516; doi:10.3389/fnins.2025.1629500)
Supplement: Supplementary file 4 [file Data_Sheet_4.pdf]

S4 Appendix. Complete Questionnaire in German.

Herzlich willkommen und vielen Dank, dass Sie sich die Zeit nehmen, an unserer Studie zu Musik und Gefühlen teilzunehmen.
Bitte nehmen Sie nur teil, wenn Sie mindestens 18 Jahre alt sind.
Die Umfrage wird ca. 15 Minuten dauern. Bitte stellen Sie sicher, dass Sie in dieser Zeit ungestört sind.
Ihre Teilnahme ist freiwillig, Sie können die Umfrage jederzeit abbrechen. Dies hat keine negativen Konsequenzen für Sie. Wir können Ihre Antworten jedoch nur auswerten, wenn Sie alle Fragen beantworten.
Ihre Antworten werden anonymisiert, können also nicht Ihrer Person zugeordnet werden.

Socio-demographic questions

Bitte beantworten Sie zuerst einige demographische Fragen.

- SD01: Wie alt sind Sie?
  - Ich bin .... Jahre alt.
- SD02: Welches Geschlecht haben Sie?
  - weiblich
  - männlich
  - divers
- SD03: In welchem Land leben Sie?
  - Ich lebe in …

Subjective social status

Im Folgenden möchten wir Sie bitten, einige Fragen zu Ihrer sozioökonomischen Situation zu beantworten.

- SE01: Verglichen mit den Bürgern Ihres Landes: Wie schätzen Sie Ihren höchsten allgemeinbildenden Schulabschluss ein?
  - Stark überdurchschnittlich
  - Etwas überdurchschnittlich
  - Durchschnittlich
  - Etwas unterdurchschnittlich
  - Stark unterdurchschnittlich
- SE02: Verglichen mit den Bürgern Ihres Landes: Wie schätzen Sie Ihren höchsten beruflichen Ausbildungsabschluss oder Hochschulabschluss ein? Falls Sie sich noch in Ausbildung bzw. Studium befinden, gehen Sie von Ihrem angestrebten Ausbildungsabschluss aus.
  - Stark überdurchschnittlich
  - Etwas überdurchschnittlich
  - Durchschnittlich
  - Etwas unterdurchschnittlich
  - Stark unterdurchschnittlich
- SE03: Verglichen mit den Bürgern Ihres Landes: Wie schätzen Sie Ihr Netto-Einkommen des Haushalts ein, in dem Sie leben? Falls Sie sich noch in Ausbildung oder Studium befinden, gehen Sie vom Netto-Einkommen Ihres angestrebten Berufes aus.
  - Stark überdurchschnittlich
  - Etwas überdurchschnittlich
  - Durchschnittlich
  - Etwas unterdurchschnittlich
  - Stark unterdurchschnittlich
- SE04: Verglichen mit den Bürgern Ihres Landes: Wie schätzen Sie den sozialen Status ihrer zurzeit ausgeübten beruflichen Tätigkeit ein? Wenn Sie mit Ihrem Partner/Ihrer Partnerin zusammenleben und diese*r einen höheren beruflichen Status als Sie hat, schätzen Sie den Status Ihres Partners/Ihrer Partnerin ein. Wenn Sie sich noch in Schule/Ausbildung/Studium

befinden, gehen Sie vom Status Ihres angestrebten Berufs aus. Im Fall der Arbeitslosigkeit gehen Sie vom Status Ihrer letzten beruflichen Tätigkeit aus.
    - Stark überdurchschnittlich
    - Etwas überdurchschnittlich
    - Durchschnittlich
    - Etwas unterdurchschnittlich
    - Stark unterdurchschnittlich
- SE05: Verglichen mit den Bürgern Ihres Landes: Wie schätzen Sie insgesamt Ihren sozialen Status ein? Falls Sie sich noch in Ausbildung oder Studium befinden, gehen Sie von Ihrem realistisch zu erwartenden sozialen Status aus.
    - Stark überdurchschnittlich
    - Etwas überdurchschnittlich
    - Durchschnittlich
    - Etwas unterdurchschnittlich
    - Stark unterdurchschnittlich

Objective socio-economic status

- SE06: Welchen höchsten allgemeinbildenden Schulabschluss haben Sie (z. B. Abitur, Realschulabschluss, noch keinen Schulabschluss)?
- SE07: Welchen höchsten beruflichen Ausbildungsabschluss oder Hochschulabschluss haben Sie?
    - Universitätsabschluss
    - Noch keinen Universitätsabschluss, aber Studium an einer Universität/Fachhochschule
    - Abgeschlossene Berufsausbildung/Lehre
    - Noch keine abgeschlossene Berufsausbildung, aber zurzeit in Ausbildung
    - keinen beruflichen Abschluss und nicht in beruflicher Ausbildung
    - zurzeit noch Schüler*in
- SE08: Wie hoch ist Ihr monatliches Netto-Einkommen? Bitte geben Sie die Währung dazu an. Wenn Sie mit Ihrem Partner/Ihrer Partnerin zusammenleben und diese*r ein höheres Einkommen hat, geben Sie das Netto-Einkommen Ihres Partners/Ihrer Partnerin an. Falls Sie sich noch in Ausbildung/Studium befinden, gehen Sie vom Einkommen des Haushalts Ihrer Eltern aus, in dem Sie leben bzw. zuletzt gelebt haben.
    - Netto-Einkommen von mir / meinem Partner / meiner Partnerin / meiner Eltern (mit Währung):
    - Möchte ich nicht angeben / weiß ich nicht

Musical sophistication

Im Folgenden werden wir Ihnen einige Statements präsentieren und einige Fragen zu Ihrem musikalischen Hintergrund, Ihren Interessen und Aktivitäten stellen. Bitte kreuzen Sie die Antwort an, die am ehesten zutrifft.

- Skala: Stimme ganz und gar nicht zu, stimme nicht zu, stimme eher nicht zu, weder noch, stimme eher zu, stimme zu, stimme voll und ganz zu
- MS02_01: Ich beschäftige mich in meiner Freizeit viel mit musikbezogenen Aktivitäten
- MS02_02: Ich schreibe gerne über Musik, z. B. in Internetblogs oder Foren.
- MS02_03: Ich lese oder suche oft im Internet nach Dingen, die mit Musik zu tun haben
- MS02_04: Ich gebe nicht viel von meinem verfügbaren Einkommen für Musik aus.
- MS02_05: Musik ist für mich eine Art von Sucht, ohne sie könnte ich nicht leben.
- MS02_06: Ich halte mich auf dem Laufenden, was neue Musik angeht (z. B. neue Künstler oder Aufnahmen).
- MS02_07: Ich suche oft Musik aus, bei der ich eine Gänsehaut bekomme.
- MS02_08: Musikstücke rufen selten Gefühle in mir hervor.
- MS02_09: Ich suche häufig eine bestimmte Musik aus, um mich zu motivieren oder zu begeistern.

- MS02_10: Ich kann identifizieren, was das Besondere an einem bestimmten Musikstück ist.
- MS02_11: Ich bin in der Lage, über meine Gefühle, die durch Musik hervorgerufen werden, zu sprechen.
- MS02_12: Musik kann bei mir Erinnerungen an Personen und Orte hervorrufen.
- MS02_13: Ich bin noch nie für meine musikalischen Fähigkeiten gelobt worden.
- MS02_14: Ich würde mich selbst nicht als Musiker/-in bezeichnen.
- MS02_15: Ich kann auswendig singen oder Musik spielen.
- MS02_16: Ich bin in der Lage, die richtigen Töne zu treffen, wenn ich zu einer Aufnahme mitsinge.
- MS02_17: Wenn jemand anders ein Lied singt, das ich kenne, kann ich eine zweite Stimme dazu singen.
- MS03 (AE_08): Ich höre jeden Tag aufmerksam Musik für …
    - 0-15 min
    - 15-30 min
    - 30-60 min
    - 60-90 min
    - 2 Std
    - 2-3 Std
    - 4 Std oder mehr
- MS04 (MT_01): Ich habe regelmäßig und täglich ein Instrument (einschließlich Gesang) für … Jahre geübt.
    - 0 / 1 / 2 / 3 / 4-5 / 6-9 / 10 oder mehr
- MS05 (MT_02): An dem Höhepunkt meines Interesses habe ich mein Hauptinstrument … Stunden pro Tag geübt.
    - 0 / 0,5 / 1 / 1,5 / 2 / 3-4 /5 oder mehr
- MS06 (MT_04): Ich habe … Jahre Unterricht in Musiktheorie (außerhalb der Schule) erhalten.
    - 0 / 0,5 / 1 / 2 / 3 /4-6 / 7 oder mehr
- MS07 (MT_05): Ich habe … Jahre Musikunterricht auf einem Instrument (einschließlich Gesang) in meinem bisherigen Leben gehabt.
    - 0 / 0,5 / 1 / 2 / 3-5 / 6-9 / 10 oder mehr
- MS08 (MT_06): Ich kann … verschiedene Instrumente spielen.
    - 0 / 1 / 2 / 3 / 4 / 5 / 6 oder mehr

Resilience

Nun möchten wir Sie bitten, noch ein paar Fragen zu Ihrer Person zu beantworten. Bitte geben Sie an, wie sehr Sie den folgenden Aussagen zustimmen.

- RS02: CD_RISC
  Skala: überhaupt nicht wahr, selten wahr, manchmal wahr, oft wahr, fast immer wahr
    - RS02_01: Ich bin fähig mich anzupassen, wenn sich etwas verändert.
    - RS02_02: Ich komme mit allem klar, was sich mir in den Weg stellt.
    - RS02_03: Wenn ich mit Problemen konfrontiert bin, versuche ich dies mit Humor zu sehen.
    - RS02_04: Der Umgang mit Stress kann mich stärken.
    - RS02_05: Ich neige dazu, mich nach Krankheit, Verletzungen oder anderen Missgeschicken wieder gut zu erholen.
    - RS02_06: Auch wenn es Hindernisse gibt, bin ich der Meinung, meine Ziele erreichen zu können.
    - RS02_07: Wenn ich unter Druck stehe, bleibe ich fokussiert und denke klar.
    - RS02_08: Wenn ich versage, lasse ich mich nicht leicht entmutigen.
    - RS02_09: Wenn es um den Umgang mit Herausforderungen des Lebens und allgemeine Schwierigkeiten geht, schätze ich mich als starke Person ein.

    - RS02_10: Ich bin fähig, mit unerfreulichen oder schmerzhaften Gefühlen wie Traurigkeit, Angst und Wut umzugehen.
- RS03: BRS
  Skala: Stimme überhaupt nicht zu, stimme eher nicht zu, neutral, stimme eher zu, stimme vollkommen zu
    - RS03_01: Ich neige dazu, mich nach schwierigen Zeiten schnell zu erholen
    - RS03_02: Es fällt mir schwer, stressige Situationen durchzustehen.
    - RS03_03: Ich brauche nicht viel Zeit, um mich von einem stressigen Ereignis zu erholen.
    - RS03_04: Es fällt mir schwer zur Normalität zurückzukehren, wenn etwas Schlimmes passiert ist.
    - RS03_05: Normalerweise überstehe ich schwierige Zeiten ohne größere Probleme.
    - RS03_06: Ich brauche tendenziell lange, um über Rückschläge in meinem Leben hinwegzukommen.

Use of music

Bitte beantworten Sie die folgenden Fragen zu Ihrem Umgang mit Musik im Alltag.

- MU02: Wenn ich Stress habe, höre oder mache ich Musik, um mich zu beruhigen oder um mich besser zu fühlen.
    - nie
    - selten
    - manchmal
    - oft
    - sehr oft
- MU03 Auf welche Art setzen Sie Musik ein, um Stress zu bewältigen? Normalerweise … (Mehrfachnennungen sind möglich)
    - MU03_1: Ich höre Musik.
    - MU03_2: Ich musiziere (z. B. Instrument, Gesang, Komponieren).
    - MU03_3: Ich tanze.
- MU04: Hatten Sie schon einmal ein emotional schwer belastendes Erlebnis in Ihrem Leben, z. B. Missbrauch / extreme Gewalt / Augenzeuge*in einer Katastrophe / Scheidung / Mobbing / schwerer Unfall / schwere Krankheit / Verlust eines nahestehenden Angehörigen?
    - Ja
    - Nein
- MU05: Wenn ja: Haben Sie nach solch einem emotional schwer belastenden Erlebnis Musik eingesetzt, z.B. um sich zu trösten, sich abzulenken oder zu motivieren?
    - Gar nicht
    - Selten
    - Manchmal
    - Oft
    - Fast immer
- MU06: Auf welche Art setzen Sie Musik ein, um emotional schwer belastende Erlebnisse zu bewältigen? (Mehrfachnennungen sind möglich)
    - MU06_1: Ich höre Musik.
    - MU06_2: Ich musiziere (Instrument oder Gesang).
    - MU06_3: Ich tanze.
- MU07: Wenn Sie nach einem emotional schwer belastenden Erlebnis Musik nutzen, wie verändert dies Ihre Stimmung? Ich fühle mich normalerweise … (Mehrfachnennungen sind möglich)
    - MU07_1: Ermutigt / aktiviert
    - MU07_2: Fröhlich / heiter
    - MU07_3: Beruhigt / friedlich
    - MU07_4: Demotiviert
    - MU07_5: Traurig
    - MU07_6: Anders; wie genau? ...

- MU08: Während der Covid-19-Pandemie nutzen einige Menschen Musik, um Ihren psychischen Stress zu reduzieren. Trifft dies auch auf Sie zu?
  - Trifft gar nicht zu
  - Trifft ein wenig zu
  - Trifft teilweise zu
  - Trifft überwiegend zu
  - Trifft voll zu

Size and weight

- MU09: Wie groß sind Sie?
  - Ich bin … cm groß.
- MU10: Wie viel wiegen Sie?
  - Ich wiege … kg.

Chronic physical illness and mental illness

- MV01: Haben Sie eine diagnostizierte chronische körperliche Krankheit?
  - Ja
  - Nein
- MV02: Wenn ja, welche?
  - MV02_01: Herz-Kreislauferkrankung
  - MV02_02: Krebs
  - MV02_03: chronische Atemwegserkrankung
  - MV02_04: Diabetes
  - MV02_05: Demenz
  - MV02_06: Autoimmunkrankheit
  - MV02_07: Chronische Rückenschmerzen
  - MV02_08: Andere
- MV03: Einige Menschen nutzen Musik, um mit ihrer chronischen Krankheit besser zurecht zu kommen, z. B., um sich zu trösten, sich abzulenken oder sich zu motivieren. Trifft dies auch auf Sie zu?
  - Trifft gar nicht zu
  - Trifft ein wenig zu
  - Trifft teilweise zu
  - Trifft überwiegend zu
  - Trifft voll zu
- MV04: Auf welche Weise nutzen Sie Musik zur Bewältigung Ihrer chronischen Krankheit? Normalerweise … (Mehrfachnennungen sind möglich)
  - MV04_01: Ich höre Musik
  - MV04_02: Ich musiziere (Instrument oder Gesang)
  - MV04_03: Ich tanze
- MV05: Wenn Sie aufgrund Ihrer chronischen Krankheit Musik nutzen, wie verändert dies Ihre Stimmung? Ich fühle mich normalerweise … (Mehrfachnennungen sind möglich)
  - MV05_01: Ermutigt/aktiviert
  - MV05_02: Fröhlich/heiter
  - MV05_03: Beruhigt/friedlich
  - MV05_04: Demotiviert
  - MV05_05: Traurig
  - MV05_06: Anders
- MV06: Haben Sie eine diagnostizierte psychische Störung, z. B. Depressionen oder eine Angststörung?
  - Ja
  - Nein

- MV07: Wenn ja, welche?

- MV07_1: Depressive Störung
- MV07_2: Bipolare Störung
- MV07_3: Angststörung
- MV07_4: Zwangsstörung
- MV07_5: Posttraumatische Belastungsstörung (PTBS)
- MV07_6: Störungen durch Substanzkonsum (z. B. Abhängigkeit von Alkohol, Cannabis, Halluzinogene, Beruhigungsmittel)
- MV07_7: Schizophrenie
- MV07_8: Angststörung
- MV07_9: Andere

- MV08: Einige Menschen nutzen Musik, um mit ihrer psychischen Störung besser zurecht zu kommen z. B., um sich zu trösten, sich abzulenken oder sich zu motivieren. Trifft dies auch auf Sie zu?
  - Trifft gar nicht zu
  - Trifft etwas zu
  - Trifft teilweise zu
  - Trifft überwiegend zu
  - Trifft voll zu
- MV09: Auf welche Weise nutzen Sie Musik zur Bewältigung Ihrer psychischen Störung? Normalerweise … (Mehrfachnennungen sind möglich)
  - MV09_01: Ich höre Musik.
  - MV09_02: Ich musiziere (Instrument oder Gesang).
  - MV09_03: Ich tanze.
- MV10: Wenn Sie aufgrund Ihrer psychischen Störung Musik nutzen, wie verändert dies Ihre Stimmung? Ich fühle mich normalerweise … (Mehrfachnennungen sind möglich)
  - MV10_01: Ermutigt/aktiviert
  - MV10_02: Fröhlich/heiter
  - MV10_03: Beruhigt/friedlich
  - MV10_04: Demotiviert
  - MV10_05: Traurig
  - MV10_06: Anders

PHQ-2

Sie haben es fast geschafft. Nun folgen noch 4 Fragen zu Ihrer Stimmung. Bitte geben Sie an, wie sehr Sie den folgenden Aussagen zustimmen.

- DE02: Wie oft fühlten Sie sich im Verlauf der letzten 2 Wochen durch die folgenden Beschwerden beeinträchtigt?
  Skala:Überhaupt nicht, an einzelnen Tagen, an mehr als der Hälfte der Tage, beinahe jeden Tag
  - DE02_01: Ich hatte wenig Interesse oder Freude an meinen Tätigkeiten.
  - DE02_02: Ich fühlte mich niedergeschlagen, schwermütig oder hoffnungslos.
- DE03: Wie oft fühlten Sie sich im Verlauf der letzten 5 Jahre an der Mehrzahl der Tage innerhalb einer Periode von mindestens 2 Wochen durch die folgenden Beschwerden beeinträchtigt?
  Skala: Nie / in einem Jahr / in mehreren Jahren / jedes Jahr
  - DE03_01: Ich hatte wenig Interesse oder Freude an meinen Tätigkeiten (in einer Periode von mindestens 2 Wochen).
  - DE03_02: Ich fühlte mich niedergeschlagen, schwermütig oder hoffnungslos (in einer Periode von mindestens 2 Wochen).

Section EN

Sie haben es geschafft, herzlichen Glückwunsch!

- EN01: Haben Sie die Fragen in dieser Umfrage gut verstanden?
  - Ja, ich habe alle Fragen verstanden.

    - Nein, ich habe ein oder zwei Fragen nicht gut verstanden. Könnten Sie angeben, welche Fragen das waren?
    - Nein, ich habe mehrere oder viele Fragen nicht gut verstanden.
- EN02: Und zuletzt: Haben Sie alle Fragen gewissenhaft und nach bestem Wissen beantwortet? Bitte beantworten Sie diese Frage ehrlich, Ihnen entstehen daraus keine Nachteile.
    - Ja, ich habe alle Fragen nach bestem Wissen beantwortet.
    - Nein.

Vielen Dank für Ihre Teilnahme und Ihre Hilfe, unsere Forschung voranzubringen.

Wichtige Anmerkungen oder Kommentare können Sie direkt an meine eMail-Adresse schreiben, indem Sie auf meinen Namen unten klicken (Andrea Bischoff).

Wenn Sie Interesse an den Ergebnissen haben, folgen Sie @Stefan Koelsch auf Twitter.

Diese Umfrage enthält Fragebogen, für die alle Rechte reserviert sind. Kein Abschnitt dieser Umfrage darf kopiert und weitergegeben werden, ohne die schriftliche Genehmigung von Prof. Dr. Stefan Koelsch (stefan.koelsch@uib.no) oder eine*r seiner Kollegen*innen, die in diesem Projekt mitarbeiten. Diese Umfrage enthält auch Fragen des Goldsmith Musical Sophistication Index (Gold-MSI), die frei erhältlich sind unter https://www.gold.ac.uk/music-mind-brain/gold-msi/.
