# Supplementary material for: The role of active music making in fostering resilience
Source: Front Neurosci. 2025 Aug 26;19:1629500. doi: 10.3389/fnins.2025.1629500 (PMC12418516; doi:10.3389/fnins.2025.1629500)
Supplement: Supplementary file 5 [file Table_1.pdf]

S1 Table. Descriptive statistics of the questionnaire measures.

Scale/subscale (number of items)	*M (SD)*	Range	Cronbach's α
Gold-MSI: all items (23)	4.42 (1.06)	1.61-6.91	.92
Active Engagement (7)	4.11 (1.14)	1.0-7.0	.82
Emotions (6)	5.56 (0.87)	1.5-7.0	.75
Musical Training (7)	3.65 (1.76)	1.0-7.0	.91
Singing Abilities (3)	4.64 (1.69)	1.0-7.0	.81
Musical anhedonia (7)	4.91 (0.97)	1.0-7.0	.75
CD-RISC (10)	2.77 (0.59)	0.2-4.0	.86
BRS (6)	3.40 (0.75)	1.0-5.0	.87
SES (5)	-2.29 (0.64)	-4.6-(-1.0)	.76
PHQ-2: major depression (2)	1.51 (1.48)	0.0-6.0	.80
PHQ-2: recurrent depression (2)	2.03 (1.96)	0.0-6.0	.93
