# Supplementary material for: The role of active music making in fostering resilience
Source: Front Neurosci. 2025 Aug 26;19:1629500. doi: 10.3389/fnins.2025.1629500 (PMC12418516; doi:10.3389/fnins.2025.1629500)
Supplement: Supplementary file 6 [file Table_2.pdf]

S2 Table. Additional information on translation, number of items and response scale format of the questionnaire measures.

Scale	Number of items	Response scale format	Translation into German	Translation into Norwegian
Gold-MSI	23	7-point Likert-Scale higher scores indicating a greater degree of musical sophistication	Schaal et al. (1)	forward-back translation of the Danish version (2) by our group
Musical Anhedonia	7	7-point Likert-Scale, higher scores indicating a lower degree of musical anhedonia	Schaal et al. (1)	forward-back translation of the Danish version (2) by our group
CD-RISC	10	5-point Likert scale, higher scores indicating greater resilience	Sarubin et al. (3)	Torgalsbøen (4)
BSR	6	5-point Likert scale, higher scores indicating greater resilience	Chmitorz et al. (5)	forward-back translation by our group
SES	5	5-point Likert scale, higher scores indicating lower subjective social status	forward-back translation by our group	forward-back translation by our group
PHQ-2	2	4-point Likert scale, higher scores indicating a greater degree of depression	Gräfe et al. (6)	forward-back translation by our group

References

1. Schaal NK, Bauer AR, Müllensiefen D. Der Gold-MSI: Replikation und Validierung eines Fragebogeninstrumentes zur Messung musikalischer Erfahrenheit anhand einer deutschen Stichprobe. Music Sci. 2014;18(4):423-447.
2. GMSI Configurator [Internet]. London: Goldsmiths University of London [cited 2024 June 11]. Available from: https://shiny.gold-msi.org/gmsiconfigurator/
3. Sarubin N, Gutt D, Giegling I, Bühner M, Hilbert S, Krähenmann O, Wolf M, Jobst A, Sabaß L, Rujescu D, Falkai P, Padberg F. (2015). Erste Analyse der psychometrischen Eigenschaften und Struktur der deutschsprachigen 10-und 25-Item Version der Connor-Davidson Resilience Scale (CD-RISC). Z Gesundheitspsychol. 2015;23(3):112-12.
4. Torgalsbøen AK. Sustaining full recovery in schizophrenia after 15 years: does resilience matter? Clin. Schizophr Relat Psychoses. 2012;5(4):193-200
5. Chmitorz A, Wenzel M, Stieglitz RD, Kunzler A, Bagusat C, Helmreich I, Gerlicher A, Kampa M, Kubiak T, Kalisch R, Lieb K, Tüscher O. Population-based validation of a German version of the Brief Resilience Scale. PloS One. 2018;13(2):e0192761.
6. Gräfe K, Zipfel S, Herzog W, Löwe, B. Screening psychischer Störungen mit dem “Gesundheitsfragebogen für Patienten (PHQ-D)“. Diagnostica. 2004;50(4):71-181.
